# Optimizing Prophylactic Antibiotic Practice for Cardiothoracic Surgery by Pharmacists’ Effects

**DOI:** 10.1097/MD.0000000000002753

**Published:** 2016-03-07

**Authors:** Ling Zhou, Jingjing Ma, Jie Gao, Shiqi Chen, Jianan Bao

**Affiliations:** From the Department of Pharmacy, The First Affiliated Hospital, School of Medicine, Soochow University, Suzhou, China.

## Abstract

Pharmacists’ role may be ideal for improving rationality of drug prescribing practice. We aimed to study the impact of multifaceted pharmacist interventions on antibiotic prophylaxis in patients undergoing clean or clean-contaminated operations in cardiothoracic department.

A pre-test–post-test quasiexperimental study was conducted in a cardiothoracic ward at a tertiary teaching hospital in Suzhou, China. Patients admitted to the ward were collected as baseline group (2011.7–2012.12) and intervention group (2013.7–2014.12), respectively. The criteria of prophylaxis antibiotic utilization were established on the basis of the published guidelines and official documents. During the intervention phase, a dedicated pharmacist was assigned and multifaceted interventions were implemented in the ward. Then we compared the differences in antibiotic utilization, bacterial resistance, clinical and economic outcomes between the 2 groups. Furthermore, patients were collected after the intervention (2015.1–2015.6) to evaluate the sustained effects of pharmacist interventions.

412 and 551 patients were included in the baseline and intervention groups, while 156 patients in postintervention group, respectively. Compared with baseline group, a significant increase was found in the proportion of antibiotic prophylaxis, the proportion of rational antibiotic selection, the proportion of suitable prophylactic antibiotic duration, and the proportion of suitable timing of administration of the first preoperative dose (*P* < 0.001). Meanwhile, a significant reduction was seen in the rate of unnecessary replacement of antibiotics and the rate of unnecessary combinations (*P* < 0.001). Besides, pharmacist intervention resulted in favorable outcomes with significantly decreased rates of surgical site infections, prophylactic antibiotic cost, and significantly shortened length of stay (*P* < 0.05). Furthermore, there were also significant decreases of the rates of antibiotic resistant *enterobacter cloacae*, *klebsiella pneumonia*, and *staphylococcus aureus* (*P* < 0.05). Moreover, the effects were sustained after discontinuation of the active interventions, as shown in prophylactic antibiotic utilization data.

Pharmacist interventions in cardiothoracic surgery result in a high adherence to evidence-based treatment guidelines and a profound culture change in drug prescribing with favorable outcomes. The effects of pharmacist intervention are sustained and the role of pharmacists is emphasized for rational medication and optimal outcomes in clinical treatment.

## INTRODUCTION

Cardiac surgery and cardiopulmonary bypass are potent inducers of a systemic inflammatory response.^1^ Surgical site infections (SSIs), particularly sterna and mediastinal infection, are associated with adverse prognosis, such as increasing morbidity and mortality. More than 50% of pathogens implicated in infections are the coagulase-positive *Staphylococcus aureus* or the coagulase-negative *Staphylococcus epidermidis*.^2^ Among different approaches to prevent SSIs, antibiotic prophylaxis is substantially important. In 2006 and 2007, the Society of Thoracic Surgeons Practice released evidence-based practice guidelines on antibiotic prophylaxis in cardiac surgery.^2,3^ Furthermore, based on the national circumstance, the National Health and Family Planning Commission (NHFPC) of China has incorporated the guidelines into the national drug policy. The official document for rational use and standard management of antibiotics,^4^ and the notice regarding national special measure scheme on clinical use of antibiotics,^5^ were issued in 2009 and 2011, respectively. The main recommendations of prophylactic antibiotic use for cardiothoracic surgery are based on the above guidelines and official documents.

Despite the availability of these guidelines, the practice of antibiotic prophylaxis is still far from optimal. A considerable portion of surgeons do not adhere to the basic principles suggested by issued guidelines.^6^ With respect to antibiotic resistance that may be caused by irrational antibiotic use, they still attributed responsibility to patients, other countries, and healthcare settings. Meanwhile, they regarded antibiotic resistance as a low priority and a distant consequence of antibiotic prescribing.^7^ As for cardiovascular surgery, the antimicrobial prophylaxis is discordance with practice standards, leading to inappropriate administration of many antibiotics.^8^ Analogously, the irrational use of antibiotics in the perioperative period of surgical procedures was ubiquitous in the department of cardiothoracic surgery of the first affiliated hospital of Soochow University, located in Suzhou, China. To prevent the complications of inappropriate administration of antimicrobials, it is quite necessary to implement interventions to improve the rationality of antibiotic prophylactic utilization.

Special pharmacists have become an established feature of the medical stewardship landscape in hospitals. As key members of the medical team, they fulfilled a vital function of overall responsibility for initiatives to promote rational drug prescribing.^9^ Discharge counseling sessions implemented by pharmacists were essential to improve outpatients’ primary medication adherence.^10^ Pharmacist interventions can facilitate knowledge translation of new evidence into practice. It has been reported that the implementation of pharmacist intervention in stress ulcer prophylaxis was associated with a decrease in inappropriate acid suppression rates during hospitalization and upon discharge, as well as significant cost savings.^11^ Furthermore, pharmacist involvement on the intensive care unit team was associated with a substantial increase in therapeutic optimization and a clinically notable reduction in preventable adverse drug events, as well as an estimated 30% increase in associated cost savings.^12^ As for antibiotic prophylaxis, pharmacist intervention promoted rational use of antibiotic with significant reduction in antibiotic costs.^13,14^ Until now, there have not been literatures about pharmacist interventions in cardiothoracic surgery wards, especially in improving rational drug utilization.

In this single-group pre-test–post–test quasiexperimental study, we compared hospital antibiotic prescribing practices pre- and during intervention. We found that the implementation of pharmacist intervention resulted in a high adherence to evidence-based treatment guidelines, with a favorable clinical and economic outcome and improved bacterial resistance. Our results highlight the role of pharmacists in rational drug prescribing practices. We believe this newly reported antibiotic pharmacist intervention may play a key role in a culture change in antimicrobial prescribing, improving the current practice and leading to favorable clinical outcomes.

## METHODS

### Population

Patients admitted to a cardiothoracic surgical ward in the first affiliated hospital of Soochow University, which is a high-volume tertiary hospital in Suzhou, China, between July 1, 2011 and December 30, 2012 (baseline group), between July 1, 2013 and December 30, 2014 (intervention group) and between January 1, 2015 and June 30, 2015 (postintervention group) were screened for eligibility. Patients were included if they had undergone cardiothoracic surgery and the wound class of the surgical operation was clean or clean-contaminated. Patients were excluded if they had undergone emergency operation or salvage operation, which was performed less than 0.5 hour after admission or decision to avoid unnecessary morbidity and death. Patients were also excluded if they were infected before the surgery.

### Ethics Statements

This study was approved by the ethical committee of the first affiliated hospital of Soochow University. Written contents were obtained from all study participants or their legal representative for the patients under guardianship.

### Criteria of Prophylactic Antibiotic Use for Cardiothoracic Surgery

The criteria of prophylactic antibiotic use for cardiothoracic surgery were established on the basis of the published guidelines and official documents as follows: evidence-based practice guideline on antibiotic prophylaxis in cardiac surgery issued by the Society of Thoracic Surgeons Practice, the official document for rational use and standard management of antibiotics issued by NHFPC, the notice regarding national special measure scheme on clinical use of antibiotics issued by NHFPC. The main criteria of prophylaxis antibiotic use for cardiothoracic surgery are shown as follows: antimicrobial prophylaxis should be given to all patients undergoing cardiothoracic surgeries; the first- or second-generation cephalosporins (cefazolin or cefuroxime) are the antibiotics of choice. Clindamycin is reserved for cases of allergy to beta-lactams and vancomycin is recommended if presumed or known methicillin-resistant staphylococcus aureus colonization is present; the duration of antimicrobial prophylactic use should not be longer than 48 hours; the timing of the first dose should be within 0.5 to 2 h prior to the skin incision. However, the timing of the first dose is at variance in China, compared with other countries, which is within 1 hour before the surgical incision.^15^ The indicators assessed as rational use of prophylactic antibiotics are judged against these criteria.

### Pharmacist Interventions

During the intervention period, there was a dedicated clinical pharmacist (LZ) in the cardiothoracic surgery ward. The interventions were endorsed by hospital and the leadership of cardiothoracic surgery department implemented from July 2013 through December 2014. The interventions consist of: participating in ward rounds and making drug treatment plans; communicating immediately with surgeons when irrational antibiotics were prescribed; providing educational sessions and handouts about antibiotic prophylaxis for medical teams, especially the surgical residents who prescribed antibiotics and the nurses who executed prescriptions; extracting the medical records and assessing their responsible use with the help of electronic auditing system; reporting the categorized data on irrational use of prophylactic antibiotics to leadership of cardiothoracic surgery department every week.

### Criteria of Surgical Site Infection

The surgical site infections for cardiothoracic surgery were defined ^16^ as surgical site associated infections occurred within 30 days for nonimplantable surgery or within 1 year for implantable surgery and one of the following criteria was satisfied: pus punctured or drained from surgical site, except drainage fluid after contaminated surgeries; purulent secretion in surgical site or fever with temperature ≥38°C, locally pain or tenderness; evidences of infection by operational, pathological, or imageological examinations; infections diagnosed by surgeons.

### Date Collection and Analysis

The data were collected from patients’ medical records, containing patients’ demographics, antibiotic selection, antibiotic utilization, and patients’ cost. The data of bacterial resistance were obtained from bacterial testing department and SSI data were obtained from the department of infection management. The data collection was conducted by a clinical pharmacist (JJM) who was blinded to the patients’ allocation status.

For comparison between the 2 phases (baseline and intervention stages, intervention and postintervention stages), data were analyzed using χ^2^ and Fisher exact tests for categorical data (sex, operation type, operative time, antibiotic prophylaxis, timing of the first dose, duration of antibiotic prophylaxis, unnecessary antibiotics combination, antibiotic selection, rational antibiotic selection, unnecessary replacement of drugs, rate of surgical site infection, bacterial resistance rates), *t* tests and Mann–Whitney *U* tests for continuous data (age, length of stay, prophylactic antibiotics cost) to assess statistical significance respectively, with *P* < 0.05 considered statistically significant. Statistical analyses were performed with SPSS 18.0 (SPSS Inc, Chicago, IL).

## RESULTS

### Study Population

We recorded 412 patients admitted to the cardiothoracic department from July 2011 through December 2012, including 292 (70.9%) patients who underwent cardiac operations, such as cardiac valve replacement (35.0%), bentall operation (1.9%), coronary artery bypass grafting (9.2%), endovascular repair (8.0%), and atrial septal defect repair (15.1%). Ninety-nine (24.0%) patients underwent thoracic operations, such as radical esophageal cancer surgery (7.8%), radical pulmonary tumor surgery (12.8%), and bullae resection (3.4%). The remaining patients (5.1%) underwent radical resection of cardiac carcinoma, exploratory thoracotomy, and so on (Table 1). Two hundred fifty-one (60.9%) operations exceeded 3 hours. The mean age of the patients was 54.8 years. Among these patients, 233 (56.6%) was male and 342 (83.0%) took antibiotic prophylaxis.

**TABLE 1:**
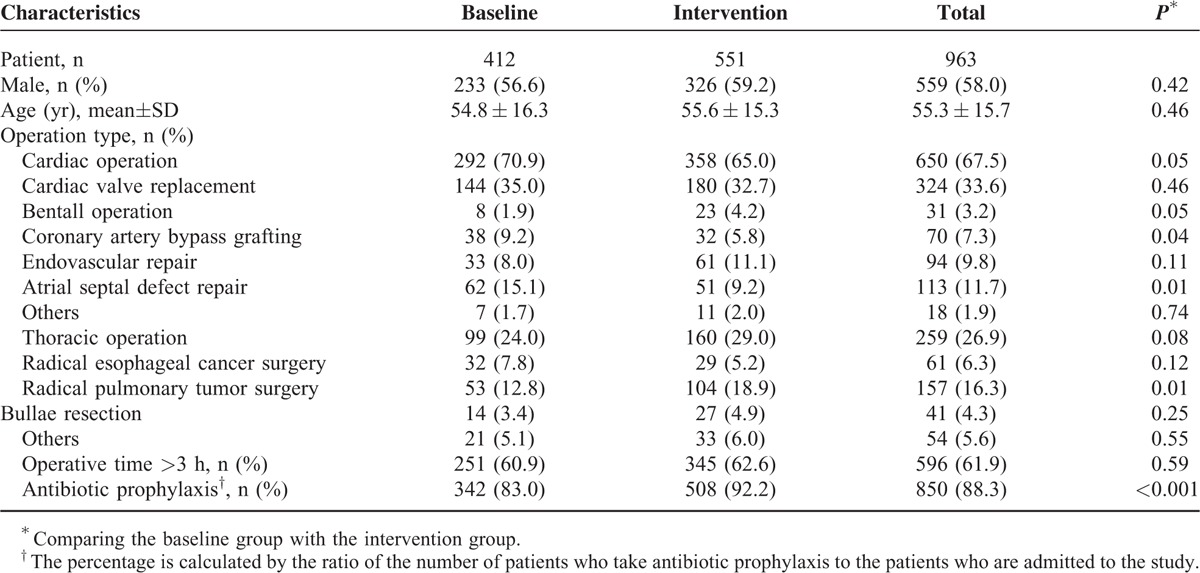
General Characteristics of Patients in the Baseline and Intervention Groups

During intervention phase, from July 2013 through December 2014, 551 patients were included. We utilized identical time period to eliminate any potential seasonal influence. The 2 groups were similar with respect to demographics and clinical characteristics (Table 1).

There was a significant increase in the proportion of patients who underwent antibiotic prophylaxis, 508 patients (92.2%) in intervention phase compared with 83.0% in baseline phase (*P* < 0.001) (Table 1).

### Rationality of Prophylactic Antibiotic Utilization Was Improved in Intervention Group

Depending on the guidelines for the rational use of antibiotic prophylaxis in the perioperative period in cardiothoracic department, we estimated the rationality of prophylactic antibiotic utilization on following aspects: the timing of the first dose, duration of antibiotic prophylaxis, antibiotic selection, antibiotic combination, and antibiotic replacement.

There was a notable increase for the first prophylactic antibiotic dose in appropriate time frame in intervention group (496, 97.6%) compared with the baseline group (157, 45.9%) (Table 2). The incorrect timing of the first dose contained more than 2 hours or less than 0.5 hour ahead of skin incision, even more, the first prophylactic dose was given after operation.

**TABLE 2:**
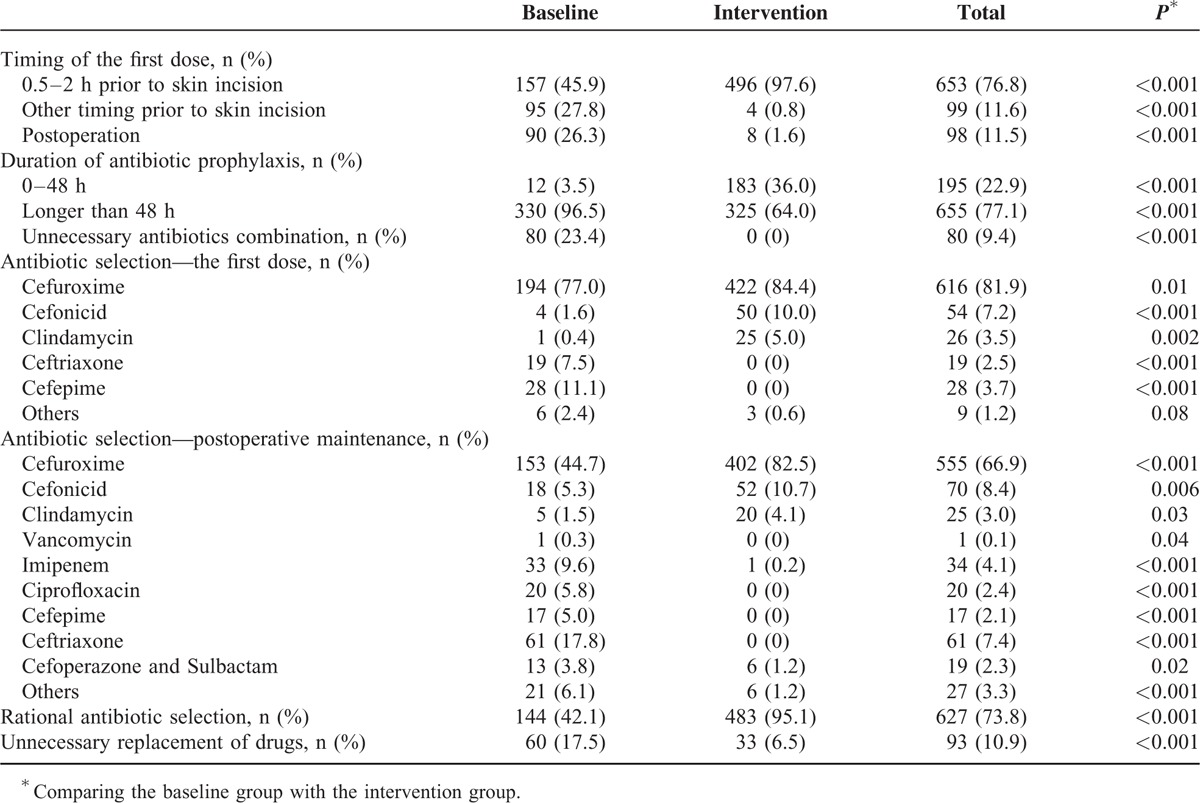
Rationality of Prophylactic Antibiotic Utilization in the Baseline and Intervention Groups

Likewise, there was a significant increase in the proportion of patients receiving antibiotic prophylaxis no more than 48 hours (*P* < 0.001). In baseline group, only 12 patients (3.5%) were given prophylactic antibiotics not more than 48 hours, while the proportion in intervention group was 36.0%. Although the improvement was obvious, the proportion was not satisfied in both groups.

As shown in Table 2, the antibiotic prescription rates of cefuroxime (*P* = 0.01), cefonicid (*P* < 0.001), and clindamycin (*P* = 0.002) were notably improved in intervention phase (84.4%, 10.0%, 5.0%) than baseline group (77.0%, 1.6%, 0.4%, respectively). Moreover, significant decrease was observed in intervention group for cefepime and ceftriaxone prescription (*P* < 0.001). As for postoperative maintenance of prophylactic antibiotics, a high rate of cefuroxime prescription (44.7%) was observed, followed by ceftriaxone (17.8%), imipenem (9.6%), ciprofloxacin (5.8%), cefonicid (5.3%), and cefepime (5.0%). Cefuroxime, cefonicid, clindamycin, and vancomycin accounted for 51.8% of the prophylactic antibiotics used. In intervention group, there was a significant increase in cefuroxime (*P* < 0.001), cefonicid (*P* = 0.006), clindamycin (*P* = 0.03) prescription, which accounted for 97.3% of the prophylactic antibiotics used. Other irrational antibiotics were all significantly decreased in intervention group (*P* < 0.01), compared with the baseline group.

We found some patients took 2 antibiotic prescriptions at the same time, which were obviously irrational utilization. In baseline group, there were 80 (23.4%) patients took 2 prophylactic antibiotics. For example, one patient was given ceftriaxone and ciprofloxacin at the same time for prophylactic purpose. During the intervention phase, this antibiotic combination was eliminated, with no person taking prophylactic antibiotic combination.

Overall, there were 483 (95.1%) patients in intervention group with rational prophylactic antibiotic selection, while there were 144 (42.1%) patients in baseline group. The difference between the 2 groups was very significant (*P* < 0.001).

Furthermore, antibiotic selection was discordant between the first dose and the postoperative maintenance in both intervention and baseline groups. Then, we found that there were unnecessary changes of prophylactic antibiotics. In baseline group, 60 (17.5%) patients replaced antibiotics unnecessarily. Compared with the baseline group, it was significantly decreased in intervention group (*P* < 0.001), with 33 (6.5%) patients replacing prophylactic antibiotics unnecessarily.

### Clinical and Economic Benefit of Pharmacist Intervention on Antibiotic Prophylaxis

To evaluate clinical effect of pharmacist intervention, we estimated the influences on SSI rates in the baseline and intervention groups (Table 3). There was a significant decrease of SSI rate (*P* = 0.02) in intervention group (3.5%) than in the baseline group (1.2%).

**TABLE 3:**
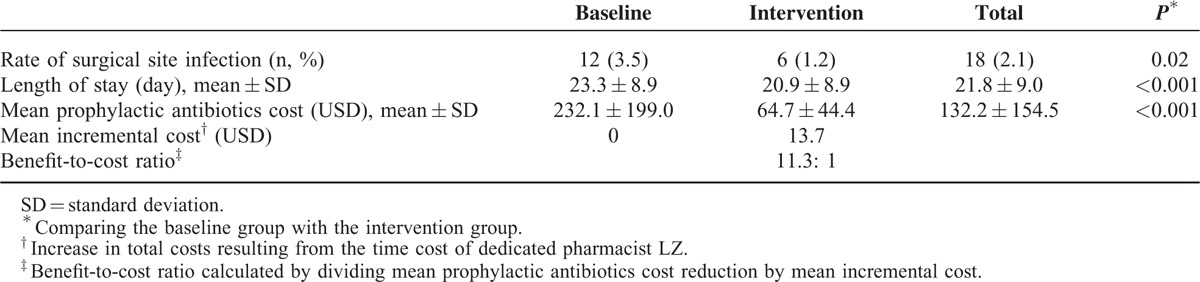
Clinical Benefit and Cost-Benefit Analysis of Pharmacist Intervention During the Baseline and Intervention Phase

Meanwhile, to evaluate economic effect, we assessed the influences of pharmacist intervention on length of stay in the baseline and intervention groups (Table 3). There was a significant decrease (*P* < 0.001) of length of stay in intervention phase (20.9 days), compared with the baseline group (23.3 days).

On the other hand, we conducted a cost-benefit analysis of pharmacist's intervention on prophylactic antibiotic practice (Table 3). There were no changes in the price of antibiotics during the intervention phase. The mean cost for antibiotic prophylaxis for those patients in baseline group was $232.1, with $64.7 in intervention group. There was a significant difference (*P* < 0.001) between the 2 groups, with the net acquisition cost reduced for antibiotic prophylaxis as $167.4. During intervention phase, the increase in total costs resulted from the time cost of dedicated pharmacist Zhou L. The benefit-to-cost ratio, calculated by dividing mean prophylactic antibiotics cost reduction by mean incremental cost, was 11.3:1 (Table 3).

### Decreased Bacterial Resistance During Intervention Phase

During baseline phase, the pathogens most commonly yielded were *klebsiella pneumonia* (22.2%), *acinetobacter baumannii* (21.4%), *enterobacter cloacae* (12.7%), and *staphylococcus epidermidis* (8.7%) among positive cultures. Then, we isolated microorganisms resistant to antibiotic agents. We found the rate of *klebsiella pneumonia* resistant to cefuroxime, cefazolin, cefotaxime, ceftriaxone, cefepime, cefoperazone, piperacillin, and trimethoprim sulfamethoxazole was 67.9%, 62.5%, 71.4%, 62.5%, 16.1%, 58.9%, 60.7%, and 66.1%, respectively. The rate of *enterobacter cloacae* resistant to cefuroxime, cefotaxime, ceftriaxone, ceftazidime, cefoperazone, piperacillin, and trimethoprim sulfamethoxazole was 68.8%, 59.4%, 53.1%, 34.3%, 56.3%, 53.1%, and 53.1%, whereas the rate of *staphylococcus aureus* resistant to trimethoprim sulfamethoxazole was 60.0%. During intervention phase, the leading pathogen positively cultured was *klebsiella pneumonia* (35.7%), followed by *acinetobacter baumannii* (21.4%), *aeruginosus bacillus* (12.1%), and *enterobacter cloacae* (8.4%). It was noteworthy that cefuroxime, cefazolin, cefotaxime, ceftriaxone, cefepime, cefoperazone, piperacillin, and trimethoprim sulfamethoxazole-resistant *klebsiella pneumonia* and cefuroxime, cefotaxime, ceftriaxone, ceftazidime, cefoperazone, piperacillin and trimethoprim sulfamethoxazole-resistant *enterobacter cloacae* were recovered during intervention phase, with the significant differences between the baseline and intervention groups (*P* < 0.05), as shown in Table 4. As for *staphylococcus aureus*, the rate of trimethoprim sulfamethoxazole resistance was significantly recovered in 14.8% (*P* = 0.02), and the rate of clindamycin resistance was decreased from 60.0% to 25.9% with pharmacist interventions, but the difference between the intervention and baseline groups was not significant (*P* > 0.05).

**TABLE 4:**
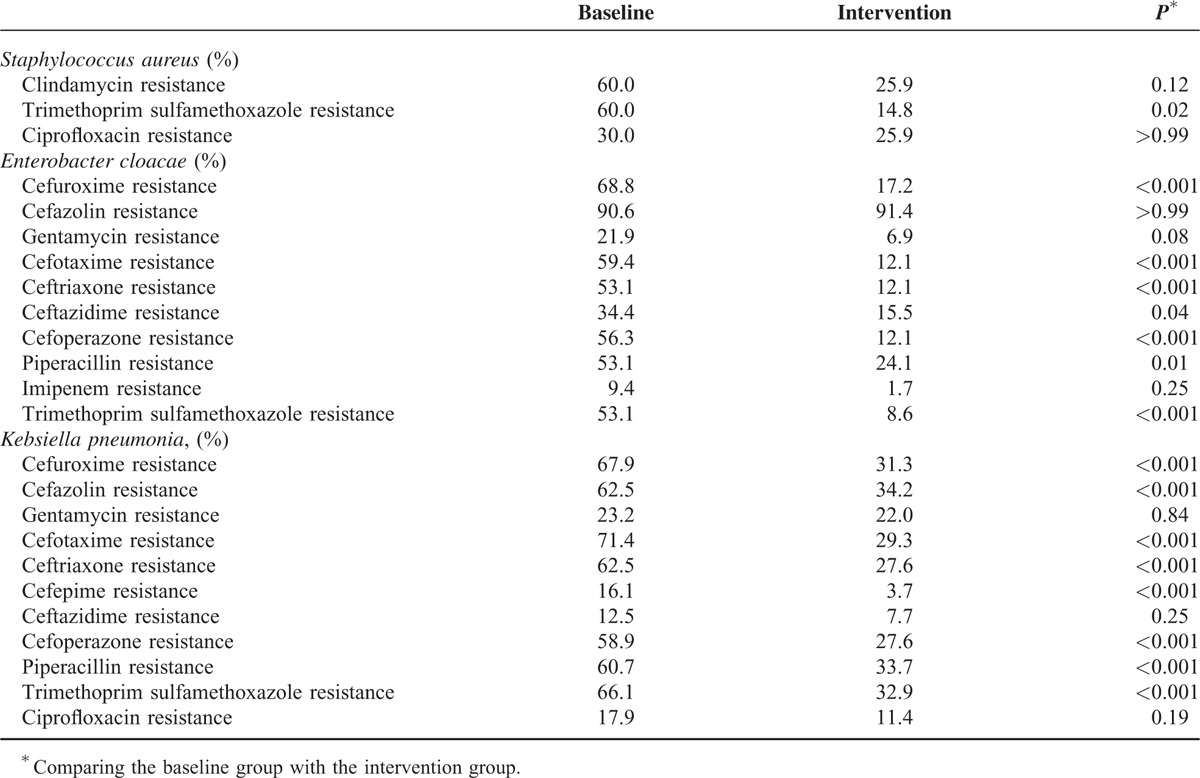
Bacterial Resistance Rates During the Baseline and Intervention Phase

### Sustained Effects of Pharmacist Intervention on Antibiotic Prophylaxis After Intervention Phase

After the interventions were finished, we further estimated prophylactic antibiotic utilization in cardiothoracic surgery for half a year (from January 2015 through June 2015) to assess the sustainable effect of pharmacist intervention. During this period, all active pharmacist interventions were discontinued and we collected the data of 156 patients admitted to the cardiothoracic department with average age of 56.8 years, including 97(62.2%) males (Table 5). The difference of sex and age between the intervention group and the postintervention group was not significant (*P* > 0.05).

**TABLE 5:**
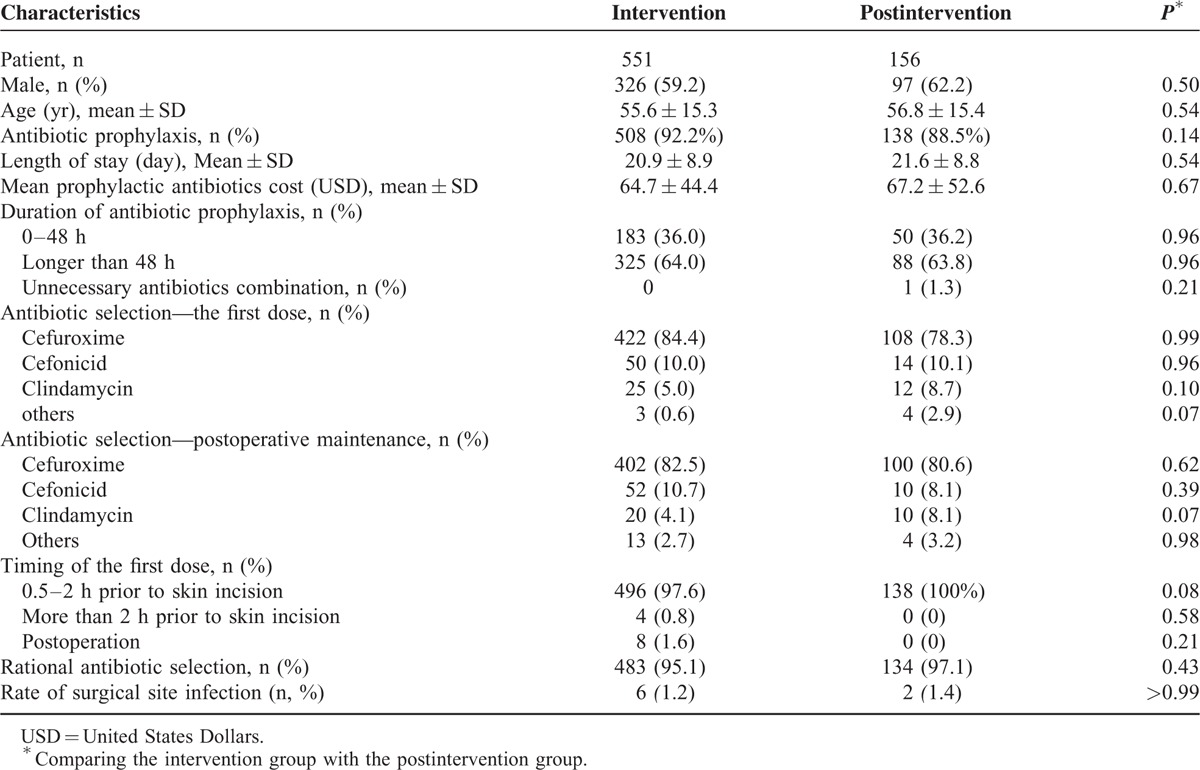
Prophylactic Antibiotic Utilization After Implementation of the Intervention Compared With During the Intervention Phase

In postintervention group, 138 (88.5%) patients received prophylactic antibiotics. There was no difference between the intervention and postintervention groups in the proportion of patient who received antibiotic prophylaxis (*P* > 0.05). With regard to economic aspects, no significant differences were found in the length of stay and mean prophylactic antibiotic cost between the 2 groups (*P* > 0.05). As for clinical outcomes, SSI rates were 1.2% and 1.4% in intervention and postintervention groups, respectively, and the difference was not significant (*P* > 0.05).

As for timing for the first dose, 138 (100%) patients in the postintervention group were treated preoperatively (0.5–2 hours) with the first dose of prophylactic antibiotics, while the proportion in the intervention group was 97.6%. Meanwhile, the difference of antibiotic combination between the 2 groups was negligible.

The analysis on the duration of antibiotic prophylactic courses yielded a similar result. A total of 36.0% and 36.2% of patients received antibiotic prophylactics for less than 48 hours in the intervention and postintervention groups, respectively.

Then we tested the rationality of antibiotic selection in the postintervention group. For the first dose, the leading antibiotics was cefuroxime (78.3%), followed by cefonicid (10.1%), clindamycin (8.7%), which were all the suitable selection. For the postoperative maintenance, the antibiotics were cefuroxime (80.6%), cefonicid (8.1%), and clindamycin (8.1%). No significant differences were found in the above antibiotic selection between the intervention and postintervention groups (*P* > 0.05). Moreover, the proportion of rational antibiotic selection was compared between the 2 groups and the difference was not significant (*P* > 0.05).

## DISCUSSION

Surgical antibiotic prophylaxis is an adjunct to surgical technique, with the goals of reducing the incidence of SSI, minimizing the effect of antibiotics on the patient's normal bacterial flora, minimizing adverse effects, and causing minimal change to the patient's host defenses.^17^ Despite the availability of practice guidelines and internal policies for perioperative prophylaxis, the compliance between prophylactic practice and national guidelines was unoptimistic and deficient.^18^ Interventions to strengthen evidence-based practice guidelines and share updated policies to surgical wards were essential. We have not found any published literatures focusing on pharmacist intervention for cardiothoracic surgery to improve prophylactic antibiotic prescription.

In this study, we implemented pharmacist intervention in cardiothoracic surgery and achieved a significant culture change in prophylactic antibiotic prescription, with favorable economic and clinical outcomes. With the help of pharmacist intervention, the proportion of antibiotic prophylaxis, appropriate choices of antibiotics, suitable duration of antibiotic prophylaxis, and the proper timing of the first preoperative dose were all improved, while unnecessary replacement of drugs or combinations were decreased. Besides, pharmacist intervention resulted in a favorable outcome with decreased SSI rate, decreased prophylactic antibiotic cost, and shortened length of stay. Furthermore, there was also positive retrieve of antibiotic resistant *klebsiella pneumonia*, *enterobacter cloacae*, and *staphylococcus* aureus. Of interest, the improvement in prophylactic antibiotic prescription and corresponding outcome was sustained after discontinuation of the active pharmacist interventions. This highlights that pharmacist interventions can trigger a sustainable change in antibiotic prophylaxis for cardiothoracic surgery.

It is reported that interventions to increase effective prescribing can improve clinical outcomes and interventions to reduce excessive antibiotic prescribing can reduce antimicrobial resistance or hospital-acquired infections.^19^ The goal of pharmacist intervention is to facilitate rational antibiotic utilization. It was reported preoperative hospital stay, age, surgeon's role, and the period of measurement were significantly associated with SSIs and after the implementation of an infection control program, SSI rate would be decreased.^20^ During the intervention phase of our study, the timing of the first dose, duration of antibiotic prophylaxis, antibiotic selection, antibiotic combination, and antibiotic replacement were all obviously improved. As a result, the SSI rate in the intervention group was decreased. SSIs are the most common reason for readmission after surgery.^21^ Besides rational antibiotic prophylaxis, appropriate hair removal, adequate maintenance of perioperative normothermia and glycaemic control should minimize this potentially preventable infection.^22^ Furthermore, antimicrobial sutures reduce the incidence of SSIs after most classes of surgery.^23^

During the intervention phase, the patients not only got optimized antibiotic prescription but also obtained economic benefits from pharmacists with the net acquisition cost reduction for antibiotic prophylaxis of $167.4 and the benefit-to-cost ratio of 11.3:1. Pharmacists improved the cost-effective use of antibiotic prophylaxis. The results were similar to the findings from other researchers. Pharmacist-managed services in people with diabetes resulted in cost saving and generated higher quality-adjusted life years with lower costs.^24^ In a pharmacist-led anticoagulation control program, the patients obtained significant increases in anticoagulation control with a minimal increase in costs.^25^ Besides, a systematic review by Gallagher et al^26^ thought clinical pharmacy interventions continued to provide cost saving and had a positive impact on hospital budgets.

Inappropriate antibiotic use is a serious public health concern, leading to bacterial resistance, increased adverse drug reactions, and risk of secondary infections, as well as a large waste of healthcare resources. Antibiotic resistance is increasing worldwide and has become a very important threat to public health and the over-consumption of antibiotics is the most important cause of antibiotic resistance.^27^ The increased prevalence of antibiotic resistance is an expected outcome of evolution. There are 2 distinct categories to deal with the new evolution: restricting the use of antibiotics and developing new ones.^28^ Pharmacist intervention in our study is one of the former- restricting the use of antibiotics. During intervention phase, bacterial resistance to several antibacterial agents was decreased. Furthermore, as Furthermore, as Riley et al^29^ presented, a new role of antibiotics was explored that crippling the pathogen while leaving the rest of the microbe largely intact.

With pharmacist intervention, length of stay of patients in cardiothoracic ward was significantly decreased. As reported, prolonged length of stay of patients undergoing cardiac surgery is associated with increased overall costs and resource consumption in addition to poorer outcomes.^30^ Interests have been heightened in controlling length of stay for hospital administrators. Ad et al^31^ thought modifiable risk factors, including lower preoperative hematocrit, higher hemoglobin A1c, major preoperative morbidity, and blood transfusion, significantly associated with length of stay in first-time cardiac surgery.

Pharmacist intervention in the study has fulfilled a vital function as key members in rational antibiotic prescription. Decades ago, the roles of antibiotic pharmacists were addressed as education staff, audit of local practices, monitoring of antibiotic consumption, participation in infection control, and so on.^32^ Several barriers to optimize pharmacist impact have been reported, including physician autonomy and limitations in the clinical training of pharmacists.^33^ Recently, Thompson et al evaluated effects of health information technology in the inpatients on mortality, length of stay, and cost. Electronic interventions were shown to have no substantial effect on these indexes.^34^ The more meaningful way for implementing intervention is essential, which puts forward the role of pharmacists. Recently, a randomized, controlled trial has been implemented to estimate the impact of pharmacist prescribing on blood pressure control in Canada with the conclusion that pharmacist prescribing for patients with hypertension resulted in a clinically important and statistically significant reduction in blood pressure.^35^ Meanwhile, a study conducted in Australia assessed whether the patients were satisfied with the pharmacist as a prescriber. They found most of the patients had a high satisfaction with pharmacist prescriber consultations and a positive outlook on the collaborative model of doctor-pharmacist prescribing.^36^ Although the generalizability of using independent pharmacist prescribing mentioned above is unknown, the results support efforts to expand pharmacists’ scope of practice in medication management activities.

In addition, we evaluated the sustained effect of pharmacist intervention after intervention phase, as enlightened by a literature about interventions of antibiotic utilization in intra-abdominal infections.^37^ There were not significant differences (*P* > 0.05) in prophylactic antibiotic usage, clinical and economic outcomes between intervention and postintervention phases.

The first dose of antibiotic prophylaxis was given during a period up to 2 hours before the first surgical incision which was indicated in internal policy. Besides, Finkelstein et al^20^ found that prophylaxis in 2 hours before surgery also reduced the risk of wound infections.

Unfortunately, the study had several limitations. Although the proportion of patients receiving antibiotic prophylaxis not more than 48 hours was increased during intervention phase, it (36.0%) was not satisfied. Another limitation of this study was the pre-to-post quasiexperimental design. This retro-prospective study was less convincing than a randomized controlled trial.^38^ However, we did not identify any other interventions during the study periods that could potentially have influenced antimicrobial use. During the whole study period, there were no new guidelines or official documents and the medical stewardship in the cardiovascular ward was the same. Even more, we also utilized identical time periods to eliminate any potential seasonal influence. In a future study, we will implement a more intrinsically rigorous design to evaluate pharmacist interventions and will take further measures to shorten antibiotic prophylactic duration.

In conclusion, our study demonstrates that real-time pharmacist intervention in cardiothoracic surgery results in a profound culture change in prophylactic antibiotic prescribing practice and a high adherence to evidence-based treatment guidelines. With the improved rationality of antibiotic utilization, favorable clinical and economic outcomes are attained and bacterial resistance is decreased. When the intervention is discontinued, the effects are still sustained. The results from this study raise the atmosphere of irrational drug utilization and emphasize the role of pharmacists in clinical treatment. In the future, more efforts are needed to expand pharmacist's practice scope to manage medication activity to address clinical inertia in prescribing habits.
